# Buffalo General Hospital

**Published:** 1858-01

**Authors:** 


*Buffalo General Hospital.*—After repeated attempts to organize such an institution, we have at least the pleasure to announce that one wing of the building of the Buffalo General Hospital is completed, and will probably be in readiness for the reception of patients on the first of May next. We have to-day been shown the plans, and there have been kindly pointed out to us, all the internal arrangements of this institution; and in convenience and beauty, we are convinced that it will be surpassed by few, if any, hospitals in America. There are some, of course, which are larger, but none which are better adapted to fulfill the objects for which this is intended. The entire structure will cost about $100,000; the wing, which is now nearly finished, and which will accommodate about one-third of the number which can be received when the entire edifice is completed, has been built at an expense of about $15,000. It is composed of a basement and two stories. The basement is cut up into kitchens, offices, rooms for the warden and physicians, etc., and the first and second stories are devoted to the sick. There are four large general wards, together with private rooms and wards, on each side of an open court: in this court is the apparatus for generating steam for heating the building, and a small engine. When this wing is in operation, as it undoubtedly will be in the spring, we will certainly have made a great and tangible advance in this public work, which has now become so desirable.

The Board of Officers is composed of Charles E. Clarke, President; Andrew J. Rich, Vice-President; William T. Wardwell, Secretary and Treasurer; and Geo. S. Hazard, Chas. E. Clarke, Andrew J. Rich, Bronson C. Rumsey, Roswell L. Burrows, William T. Wardwell, Geo. Howard, S. W. Howell and Henry Martin, Trustees. The medical staff will consist of two consulting surgeons, two consulting physicians, two attending surgeons and two attending physicians; there will also be appointed by the medical staff, two or more resident physicians and surgeons.

The by-laws have been carefully penned, and are in every way calculated to render this one of the best regulated, as well as one of the most convenient and elegant hospitals in the country. F.

